# History of in vitro fertilization and related techniques in Iran: The view of professor Safaa Al-Hasani

**DOI:** 10.18502/ijrm.v19i1.8383

**Published:** 2021-01-25

**Authors:** Abbas Aflatoonian

**Affiliations:** Research and Clinical Center for Infertility, Yazd Reproductive Sciences Institute, Shahid Sadoughi University of Medical Sciences, Yazd, Iran.

**Figure 1:**
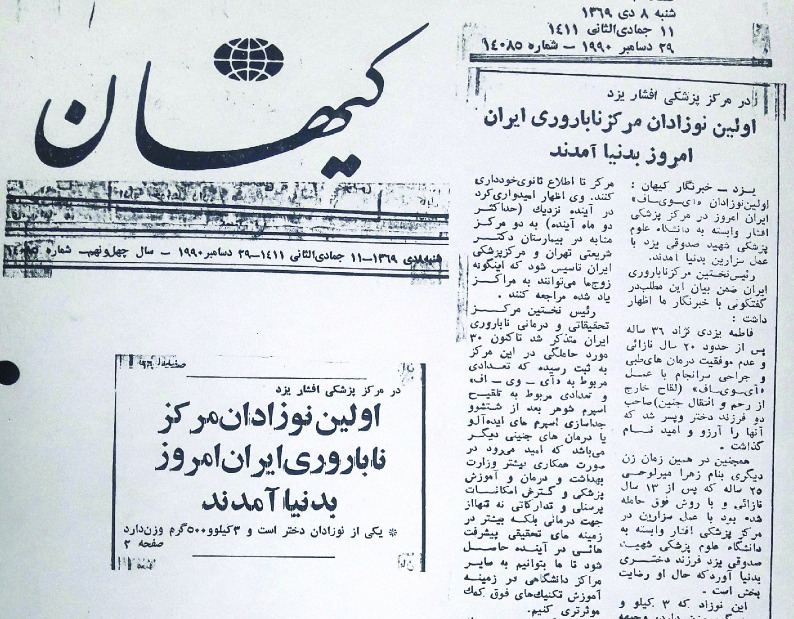
*Kayhan* newspaper reports (December 29, 1990) on the birth of first Iranian IVF babies in Yazd, Iran.

**Figure 2:**
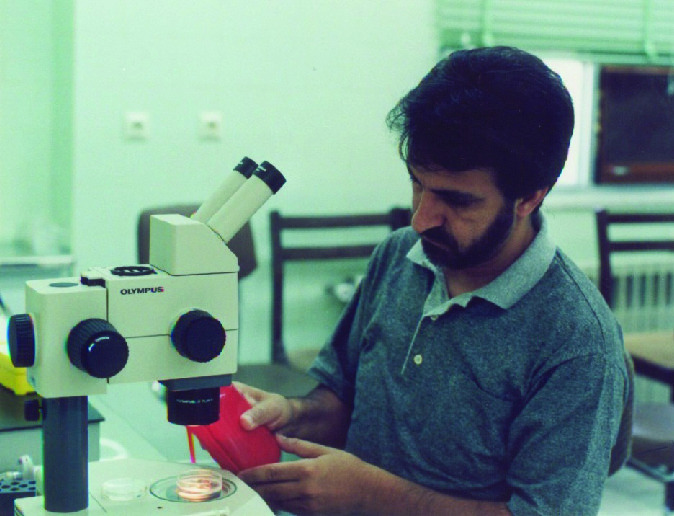
Professor Safa Al-Hasani who has contributed in establishing embryology facilities in Iran.

Life is a beautiful thing and it would be doomed without the reproductive capability of humans. In that, the assisted reproductive techniques (ART) can help infertile couples to enjoy their lives and fill in the gap. With the birth of Louise Brown, the world celebrated the beginning of a new era – an era of ART. Thirty years ago, the first Iranian IVF baby was born in Yazd (on December 28, 1990) (Figure 1). Before reaching this milestone, I had requested Professor Hammarberg and Professor Trounson, whom I had met at the 6${}^{\mathrm{th}}$ World Congress on Human Reproduction in Tokyo in 1987, to help us establish the first IVF center in Iran. However, my request was rejected at the time because of political reasons. However, Professor Safaa Al-Hasani (Figure 2) came to our rescue and helped put up the foundation of the first Iranian IVF center in Yazd, following which a few other centers in other cities of the country were also set up. Because some distorted accounts (1, 2) have published about the history of IVF in Iran, I took it upon myself to present to you this text in Professor Al-Hasani's own words. In fact, I cannot think of a better person to recount an impartial version of the events because his contributions have played a key role in the initiation of IVF in Iran.

Some details of the legend in Professor Al-Hasani's own word are as follows:

“My first trip to Iran was at the end of 1981 during the Iran–Iraq war. I met Iraqi doctors from Dawa party who left Iraq before the war in Dolat Abad, Tehran, Iran. I told them about my specialty in IVF and offered them my help to establish it in Tehran. Their priority at the time was the war against the aggressor, Sadam Hussain, and they told me that they had to concentrate on the same.

In June 1983, I met Dr. Mohammad Memarzadeh in a congress in Dublin, Ireland. I was so happy to meet an Iranian doctor at that occasion. I told him about my trip to Tehran in 1981 and explained to him our IVF results from Germany.

At the end of 1983, I took part in a medical congress in Tehran. I submitted my abstract about IVF, which was not accepted. However, they did invite me to take part in the medical congress.

In the year 1986, I met Dr. Mohammad Memarzadeh again in Singapore at the Fertility and Sterility Congress. We spoke about IVF, and he expressed his interest in establishing the IVF technology in Tehran. He invited me to attend a congress the same year in Tehran. This congress was organized by Jahad Danishkahy. The president of Jahad Danishkahy was Mohammad Mehdipour at the time. I went to Tehran to participate in this congress, and I spoke about the IVF technology. They were very interested in my presentation about my experience in IVF technology in Germany. I explained to them that setting up the technology was very easy and could be done at a low cost.

In the year 1987, Dr. Mohammad Memarzadeh once again invited me to a medical congress in Tehran; this congress was also being organized by Jahad Danishkahy. The topic of the congress was infertility and IVF. I prepared a 20-min lecture to present, which was confirmed by Dr. Memarzadeh. A few foreign-language speakers also attended the congress. However, 10 mins into my presentation, the head of that session asked me to stop. I was annoyed and told him that I had 20 min to present my lecture, which was also confirmed by Dr. Memarzadeh. He, however, asked me to stop. Consequently, I asked him why they had invited me from Germany when they were not interested in listening to my lecture. I told them they could have rather saved my travel cost for the poor people in Iran. I left my presentation and went back to my seat. Subsequently, a doctor came to me and asked me if I could travel to another city with him for one day. He told me that they had bought all the necessary equipment from Germany. He asked me to help them. I happily agreed. This doctor was Dr. Abbas Aflatoonian from Yazd City. He finished his presentation on Laparoscopy and took me to the airport on the same day. I left the congress because I wanted to help any center in Iran that was interested in establishing the IVF technology. I felt that this was my duty as a Muslim.

I went to Yazd with Dr. Abbas Aflatoonian the same day. At the airport, we met the Dean of the Yazd University, who spoke with me in Arabic and said, "You are welcome in your second home." It was a huge pleasure for me. The name of this doctor was Dr. Faqihi. I inspected the center (Safayieh Fertility Center), which was interestingly very suitable for treating infertile patients. It was located in a beautiful place and designed very nicely.

I gave several lectures about the procedures of IVF until midnight. I promised Dr. Aflatoonian and his colleagues to come back to Yazd after one month to start the treatment of the patients. I told them that some equipment from the Labotect Company were not suitable to work with. I asked if they had a machine that had a constant 37ºC. They brought it from the store and it looked like it was made in Iran. We put it inside a desiccator and inside it a tube of CO${}_{2}$. We checked the PH of the media through the color. I told them the incubator was ready for work. In the same year, I came back to Yazd City with culture media and disposable material. Dr. Abbas Aflatoonian prepared patients for treatment. We received good-quality embryos and were able to achieve pregnancy using this simple way. This success made me very happy as I could establish for the first time the IVF procedure in the Islamic Republic of Iran. Not to forget, my duty was also to help the poor people in this country. I asked the team in this center to keep the treatment cost fairly low, and if they did that, I promised to supply them with all disposable material from Germany because they could not get it themselves from abroad. Thus, the price was fixed at 5,000 Tomans. After the success, I suggested having more centers in Iran; the idea being one center for each one million inhabitants. If there was only one center, that is, the Yazd Center at the time, the patients would come from the entire country for treatment and the center would not have the capacity to provide quality services to them. Initially, more than 5,000 couples came to be treated. There were no hotels in the city and the patients slept in the street. My team at the center learnt the procedure very quickly. Mr. Amir Arjmand, and later Mr. Ali Ahmadi and Mr. Mehrdad Solymani worked in the laboratory, while Dr. Abbas Aflatoonian, Dr. Mohammad Ali Karimzadeh Meybodi, Dr. Syed Mohammad Kazemini, and later Dr. Serajoddin Vahidi were the doctors. I visited Yazd three to four times every year. After the success, the Deputy Minister of Research and Technology of the Ministry of Health (Dr. Masih Hashemy at the time) asked me to visit the centers in Tehran. They gave me a car with a driver to transfer me from one center to another. The first center was in the Dr. Ali Shariaty Hospital (the doctors were Dr. Agha Hossaini, Dr. Alyassin, and Dr. Saidi; and Mr. Hujattollah Saidi in the laboratory). The next center was Royan Institute (the doctors were Dr. Ashrafi and Dr. Moeini; and Dr. Ahmad Hossaini, Dr. Mohammad Mehdi Akhondi, and Dr. Mojtaba Rezazadeh in the laboratory).

I have visited a private center in the Aban Hospital where Dr. Saremi worked with Mr. Saidi in the laboratory, once in 1988 and again at the end of 1989. The Ministry of Health invited me and my family to visit Yazd and Tehran. We were sitting in the airplane, ready to fly back to Germany, when the news came from our work that the first Iranian babies (one single and one twin) had been born in Yazd.

Then, in 1991, I was called at the Iranian embassy in Bonn to receive money from the Ministry of Health. I was handed over a check of 98,000 German Mark. I asked Dr. Masih Hashmy what the money was for. He asked me to buy equal equipment for the laboratories in Shariaty Hospital and Yazd University. I gave orders to one of the export companies in Germany.

**Figure 3:**
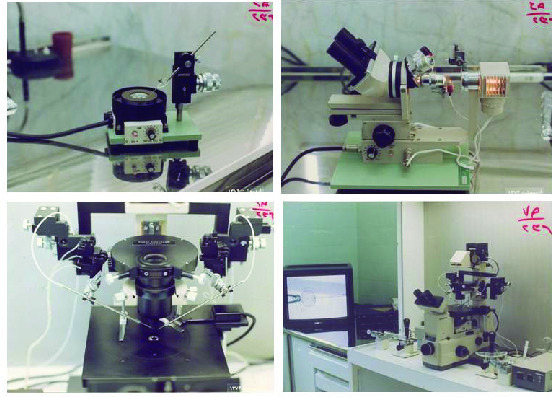
Facilities for performing ICSI in Yazd in 1993.

They delivered the equipment as I had asked. By the end of 1993, the new method of infertility treatment, the ICSI method, was established for treating severe male infertility (Figure 3).

I went to Yazd to establish this method. We received good results. Later, I went to Shariaty Hospital. They prepared 24 patients for one day, six of whom became pregnant. In addition, I established the same method in Royan Institute. In 1994, I was invited with my family to the Holy City of Mashhad. We established a new center there. The Yazd IVF Center organized many workshops and congresses. In 2004, a new method came for cryopreservation. Again, I went to Yazd to establish this method. A workshop was also organized and many embryologists took part in this event. Now, the number of IVF centers has increased rapidly and more than 80 centers are available in Iran. The scientific level of these centers has become the highest in the region. Also, in many international congresses, the number of abstracts submitted from Iran is more than some European countries. The research in Royan Institute and other universities has met worldwide acceptance.

I am so happy now that I could serve the Islamic Republic of Iran and to see the high- standard Reproductive Medicine in Iran.”
